# The Functional Deubiquitinating Enzymes in Control of Innate Antiviral Immunity

**DOI:** 10.1002/advs.202002484

**Published:** 2020-12-15

**Authors:** Zhi Zong, Zhengkui Zhang, Liming Wu, Long Zhang, Fangfang Zhou

**Affiliations:** ^1^ Department of Hepatobiliary and Pancreatic Surgery The First Affiliated Hospital Zhejiang University School of Medicine Hangzhou 310003 P. R. China; ^2^ MOE Key Laboratory of Biosystems Homeostasis & Protection and Innovation Center for Cell Signaling Network Life Sciences Institute Zhejiang University Hangzhou 310058 P. R. China; ^3^ Institute of Biology and Medical Science Soochow University Suzhou 215123 P. R. China

**Keywords:** deubiquitinating enzymes, innate antiviral immunity, pattern recognition receptors, therapeutic approach

## Abstract

Innate antiviral immunity is the first line of host defense against invading viral pathogens. Immunity activation primarily relies on the recognition of pathogen‐associated molecular patterns (PAMPs) by pattern recognition receptors (PRRs). Viral proteins or nucleic acids mainly engage three classes of PRRs: Toll‐like receptors (TLRs), retinoic acid‐inducible gene I (RIG‐I)‐like receptors (RLRs), and DNA sensor cyclic GMP‐AMP (cGAMP) synthase (cGAS). These receptors initiate a series of signaling cascades that lead to the production of proinflammatory cytokines and type I interferon (IFN‐I) in response to viral infection. This system requires precise regulation to avoid aberrant activation. Emerging evidence has unveiled the crucial roles that the ubiquitin system, especially deubiquitinating enzymes (DUBs), play in controlling immune responses. In this review, an overview of the most current findings on the function of DUBs in the innate antiviral immune pathways is provided. Insights into the role of viral DUBs in counteracting host immune responses are also provided. Furthermore, the prospects and challenges of utilizing DUBs as therapeutic targets for infectious diseases are discussed.

## Graphical abstract

This review systematically summarizes the role and mechanisms of deubiquitinating enzymes (DUBs) in regulating innate antiviral signaling via the Toll‐like receptors (TLRs), RIG‐I like receptors (RLRs), and cyclic GMP‐AMP synthase (cGAS). The role of viral DUBs counteracting host immune responses is also concluded. Moreover, the prospects and challenges of utilizing DUBs as therapeutic targets for treatment of infectious diseases like COVID‐19 are also discussed.

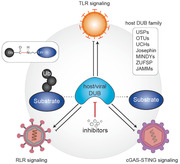

## 1 Introduction

Our living environment is surrounded by various infectious viruses. To counteract these pathogens, hosts have evolved diverse innate immune systems. This is achieved by pattern recognition receptors (PRRs) expressed in cells that recognize molecules specific to the pathogens, namely pathogen‐associated molecular patterns (PAMPs).^[^ ^1^, ^2^ ^]^ Among variety of viral PAMPs, viral proteins or nucleic acids are sensed mainly by three PRRs: Toll‐like receptors (TLRs), cytosolic RIG‐I like receptors (RLRs), and cytosolic DNA sensors, among which cyclic GMP‐AMP synthase (cGAS) is the predominant sensor of various DNA viruses.^[^ ^3^ ^]^ Upon recognition, PRR initiates a series of signaling cascades that ultimately lead to the synthesis and secretion of type I interferons (IFN‐I) and proinflammatory cytokines, thereby exhibiting antiviral immune responses.^[^ ^4^ ^]^

As insufficient interferon production results in chronic infection whereas excessive interferon leads to autoimmune and/or inflammatory disease, innate immunity must be tightly regulated to efficiently respond to invading pathogen and meanwhile avoid excessive harmful immune pathology.^[^ ^5^ ^]^ This raises the question of how host cells limit innate immune signals to extremely low levels and meanwhile ensure its timely activation to deal with the challenge of pathogens. The rapid switch on of innate antiviral signaling requires certain posttranslational modifications (PTMs) of crucial sensors for viral RNA, such as ubiquitination.^[^ ^6^ ^]^

**Figure 1:**
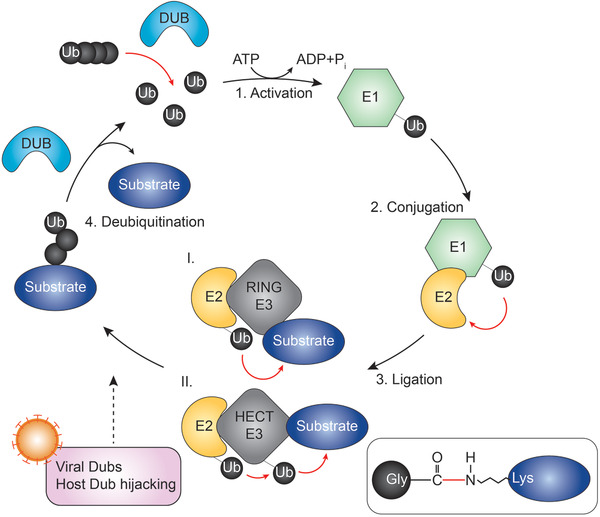
Summary of the ubiquitination cascade. Schematic of key events in ubiquitination and deubiquitination. Ubiquitin is activated by the E1‐activating enzyme in an ATP‐dependent manner. Ubiquitin then transfers to the E2‐conjugating enzyme. The E3 ligase transfers the E2‐bound ubiquitin directly (RING E3 ligase) or indirectly (HECT E3 ligase) to a substrate, forming an isopeptide bond between the carboxyl group of ubiquitin's glycine and the epsilon‐amino group of the substrate's lysine. DUBs remove ubiquitin molecules from substrates.

Ubiquitination is an enzymatic posttranslational modification in which a ubiquitin protein with 76 amino acids is conjugated to a target molecule. Ubiquitin is so named because it occurs ubiquitously in cellular processes. Ubiquitination requires a cascade consisting of three enzymes: ubiquitin‐activating enzymes (E1), ubiquitin‐conjugating enzymes (E2), and ubiquitin ligases (E3). In this cascade, E1 can bind with many E2s, which further bind with hundreds of E3s in a hierarchical way.^[^ ^7^ ^]^ To date, three classes of E3s have been identified: really interesting new gene (RING), homologous to E6AP C‐terminus (HECT), and RING‐between‐RING (RBR). RING E3s directly transfer the ubiquitin from E2s to the substrate, whereas HECT and RBR E3s first receive ubiquitin from E2s through a catalytic cysteine, after which the ubiquitin is transferred to the substrate.^[^ ^8^ ^]^ An isopeptide bond is then formed between the carboxyl group of the ubiquitin's glycine and the epsilon‐amino group of the substrate's lysine^[^ ^7^ ^]^ (**Figure** **1**). It is worth mentioning that some E3s confer substrate specificity, generally through their protein–protein interaction domains or binding motifs.^[^ ^8^ ^]^ Ubiquitin has seven lysine residues (K6, K11, K27, K29, K33, K48, and K63) and an N‐terminus methionine (M1) serving as points of ubiquitination, thereby resulting in complex linkage types.^[^ ^7^ ^]^ A substrate protein can be attached with a mono‐ or poly‐ubiquitin molecule.^[^ ^7^ ^]^ The resulting ubiquitinated proteins vary based on the linkage type of the ubiquitin chains.^[^ ^9^ ^]^ The most common linkage types are K48‐ and K63‐linked polyubiquitin chains, with the former recognized by the proteasome, leading to the degradation of the substrate, whereas the latter is mainly involved in nondegradative roles, including cellular signaling, intracellular trafficking, and DNA damage response.^[^ ^10^, ^11^, ^12^ ^]^ Those not linked via canonical K48 linkages or K63 linkages are known as atypical ubiquitin chains, whose physiological roles have only started to accumulate.^[^ ^13^ ^]^ For example, linear ubiquitin chains are linked through M1 and have been established to play crucial roles in inflammatory signaling and apoptotic cell death.^[^ ^14^ ^]^ Accumulating evidence has uncovered the role of both typical and atypical ubiquitin chains in the initiation, maintenance, and termination of antiviral immune responses.^[^ ^15^, ^16^ ^]^

Ubiquitination is a dynamic and reversible reaction. Its opposing process is termed deubiquitination, a process that is performed by another set of over 100 enzymes, namely deubiquitinating enzymes (DUBs).^[^ ^17^, ^18^ ^]^ DUBs cleave ubiquitin molecules from substrates to generate a free ubiquitin pool, thereby ensuring the ubiquitin cycle^[^ ^19^ ^]^ (Figure 1). Fine‐tuning of antiviral immunity requires the balance between the addition and removal of ubiquitin moieties. In this Review, we introduce the basic knowledge of DUBs and summarize the mechanisms and roles of DUBs in control of three major innate antiviral immune pathways: the TLR, RLR, and cGAS signaling. In particular, we highlight the advances in virus‐encoded DUBs that counteract host immune responses. Finally, we conclude with the mechanisms of DUBs function and discuss the prospects and obstacles involved in developing DUB inhibitors for disease treatment. This review not only extends the body of knowledge regarding the role that DUBs play in regulating host innate immune signaling pathways in viral infections, but also describes how targeting DUBs could be utilized to develop DUB inhibitors that could be applied to yield therapies for clinically relevant viral infections.

## 2 DUBs

### 2.1 Classification of DUBs

**Figure 2:**
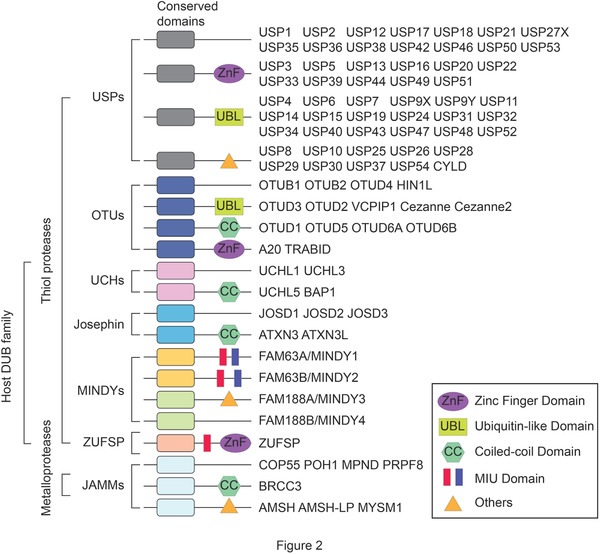
Classification and domain architecture of the host DUB family. The host DUB family can be divided into six families of cysteine proteases, namely ubiquitin‐specific proteases (USPs), ovarian tumor proteases (OTUs), ubiquitin C‐terminal hydrolases (UCHs), the Josephin family, the motif interacting with ubiquitin (MIU)‐containing novel DUB family (MINDYs), zinc finger with UFM1‐specific peptidase domain protein/C6orf113/ZUP1 (ZUFSP), and one family of metalloprotease group, namely the JAB1/MPN/MOV34 metalloenzyme family (JAMMs, also known as MPN+). The conserved and specific domains are indicated by different shapes and colors.

The number of DUBs in different organisms varies, with ≈100 enzymes occurring in humans.^[^ ^20^ ^]^ To date, DUBs have been divided into seven families, based on their structural features: six families of cysteine proteases, namely ubiquitin‐specific proteases (USPs), ovarian tumor proteases (OTUs), ubiquitin C‐terminal hydrolases (UCHs), the Josephin family, the motif interacting with ubiquitin (MIU)‐containing novel DUB family (MINDYs), zinc finger with UFM1‐specific peptidase domain protein/C6orf113/ZUP1 (ZUFSP), and the JAB1/MPN/MOV34 metalloenzyme family (JAMMs, also known as MPN+), which belongs to the zinc‐metalloprotease group.^[^ ^20^, ^21^, ^22^ ^]^ DUBs generally contain a catalytic domain surrounded by one or more accessory domains, some of which contribute to target recognition. These additional domains include zinc finger (ZnF) domain, ubiquitin‐like domain (UBL), coiled‐coil (CC) domain, and motif interacting with ubiquitin (MIU) domain (**Figure** **2**). DUBs of cysteine proteases generally contain catalytic dyads or triads (either two or three amino acids) to catalyze the hydrolysis of the amide bonds between ubiquitin and the substrate. In contrast, metalloproteases coordinate zinc ions with histidine, aspartate, and serine residues, which activates water molecules and enables them to attack the isopeptide bond.^[^ ^23^, ^24^ ^]^

### 2.2 DUB Binding and Cleavage

DUBs cleave ubiquitin from ubiquitinated substrates in different ways, either by binding to a protein substrate that they deubiquitinate (target‐specific) or by directly binding to the ubiquitin chain that they cleave (linkage‐specific).^[^ ^25^ ^]^ In the case of target‐specific manner, many DUBs directly bind target proteins. However, apart from the catalytic domains, some DUBs, such as USPs, contain additional protein–protein interaction domains that recruit protein substrates for deubiquitination.^[^ ^26^ ^]^ This allows DUBs to selectively regulate cellular processes. Notably, most USPs remove ubiquitin regardless of linkage type.^[^ ^27^ ^]^ In contrast, some DUBs target specific chain types to execute their function. For example, the JAMM metalloprotease associated molecule with the SH3 domain of STAM (AMSH) was the first identified DUB to exhibit linkage specificity, and many JAMMs are reported to be K63 specific.^[^ ^28^, ^29^ ^]^ MINDY DUBs are K48 specific.^[^ ^21^ ^]^ Most OTU family DUBs can be linkage specific,^[^ ^30^ ^]^ whereas most USPs show little or no linkage preference.^[^ ^27^, ^31^ ^]^ The underlying mechanisms of linkage specificity have been reviewed elsewhere^[^ ^25^ ^]^ and will therefore not be covered herein.

Ubiquitin chains are cleaved by DUBs either through endo‐ or exocleavage activity. While endo‐cleavage activity efficiently removes ubiquitin chains from a substrate, the released chain undergoing further processing to regenerate monoubiquitin, exo‐cleavage directly generates monoubiquitin, but requires multiple steps for the DUB to remove the entire polyubiquitin chain. Although it seems complicated to determine whether a DUB cleaves with endo‐ or exocleavage activity, several studies have elucidated that the activity depends on both the DUB structure and the ubiquitin linkage type.^[^ ^25^ ^]^

### 2.3 Mechanisms of DUB Regulation

Dysregulation of DUBs may lead to aberrant functions that can cause a variety of diseases, such as cancer,^[^ ^32^ ^]^ inflammatory diseases,^[^ ^33^ ^]^ and neurological diseases.^[^ ^34^, ^35^ ^]^ DUBs can be regulated by a multifaceted approach, including the control of their abundance, catalytic activity, as well as their localization.^[^ ^36^ ^]^

The abundance of DUBs is modulated at the transcriptional level and the expression level of DUBs can be induced in a stimulation‐dependent manner. For example, the abundance of A20 is low in unstimulated cells, yet is upregulated in response to TLR4‐mediated nuclear factor *κ*B (NF‐*κ*B) activation.^[^ ^37^, ^38^ ^]^ Once expressed, DUBs can also be negatively regulated, as exemplified by the stimulation of the expression of OTUD‐6B in B lymphocytes by IL‐3, IL‐4, IL‐13, or granulocyte‐macrophage colony‐stimulating factor (GM‐CSF); however, an opposing effect occurs with sustained induction, leading to a decrease in OTUD‐6B expression.^[^ ^39^ ^]^

DUB catalytic activity can be regulated in a number of ways: binding to regulatory proteins, allosteric regulation, and posttranslational modification. First, DUBs can target a specific substrate by recruiting other factors, as demonstrated by USP10 recruiting and interacting with the monocyte chemotactic protein induced protein 1 (MCPIP1) to deubiquitinate its substrate, nuclear factor *κ*B essential modulator (NEMO).^[^ ^40^ ^]^ Second, DUB activity is commonly adjusted in an allosteric fashion. This occurs especially when DUBs function in complexes with other molecules. For instance, USP1 by itself is an ineffective enzyme, but its activity is boosted when bound to USP1‐associated factor 1 (UAF‐1) and it undergoes conformational changes.^[^ ^41^ ^]^ Additionally, substrate binding may promote DUB activation, thereby enabling fine‐tuning of linkage specificity. For example, USP7 alters its inactive configuration toward an active one upon ubiquitin binding, which enables full activation of the USP7 catalytic domain.^[^ ^42^ ^]^ Third, DUB catalytic activity can be modulated by posttranslational modifications, such as phosphorylation, ubiquitination, and SUMOylation.^[^ ^25^, ^43^ ^]^ For instance, our previous studies demonstrated that poly‐SUMOylation is required for OTUB2 to interact with and deubiquitinate Yes‐associated protein (YAP) and transcriptional coactivator with PDZ‐binding motif (TAZ).^[^ ^44^ ^]^ Intriguingly, these modifications can play dual roles in DUB activity, either inhibitory or activating, thereby revealing the fascinating intricacy of posttranslational regulation. Moreover, the cellular microenvironment can also influence DUB activity. DUBs that are cysteine proteases harbor a reactive cysteine residue that can be oxidized by reactive oxygen species (ROS), thus attenuating DUB activity.^[^ ^45^, ^46^, ^47^ ^]^

DUB regulation is also exerted through subcellular localization, which is necessary for substrate accessibility. DUBs are often found to form complexes with other proteins, including adaptors and scaffolds. Interactions with these proteins can assure proper localization of the DUB so that the specific substrate is delivered to the DUB in the right place for its catalytic action.^[^ ^48^ ^]^ DUB localization can be regulated by various approaches. Posttranslational modifications, apart from affecting DUB catalytic activity, have also been reported to affect the nuclear localization of DUBs.^[^ ^49^, ^50^, ^51^, ^52^, ^53^ ^]^ To illustrate, phosphorylation of USP4 by protein kinase B (PKB, also known as AKT) results in its redistribution from the nucleus to the cytoplasm, which enables USP4 to reach the cell membrane and deubiquitinate the transforming growth factor‐*β* (TGF‐*β*) receptor I (T*β*R‐I).^[^ ^52^ ^]^ Furthermore, localization can also be indirectly modulated by changing the protein interactions that a DUB engages in ref. ^[^ ^25^ ^]^. For example, hydroxylation of OTUB1 by factor inhibiting HIF (FIH) leads to a profound change in the interaction of OTUB1 with proteins important in cellular metabolism.^[^ ^54^ ^]^ This alteration of OTUB1 interactome affects OTUB1 localization and substrate accessibility.

The diversity of the mechanisms for DUB regulation enables their function to be finely tuned, which ensures appropriate responses in different contexts, including innate immune response against viruses.

## 3 DUBs in PRR Signaling

### 3.1 DUBs in TLR Signaling

As the most widely studied PRRs, TLRs are transmembrane glycoproteins that are mainly expressed in immune cells. TLRs were first acknowledged to be critical for defending fungal infections.^[^ ^55^ ^]^ TLRs have evolved to recognize a variety of PAMPs.^[^ ^56^ ^]^ To date, at least ten human TLRs have been identified, with TLRs 1, 2, 4, 5, 6, and 10 localizing at the cell surface, whereas TLRs 3, 7, 8, and 9 are anchored to endolysosomes.^[^ ^57^ ^]^ Of the identified TLRs, several have been linked to innate antiviral immunity. Among them, TLR2 and TLR4 sense both structural and nonstructural viral proteins, whereas TLR3, TLR7/8, and TLR9 detect virus‐derived dsRNA, ssRNA, and unmethylated CpG DNA, respectively, which together is responsible for the effectiveness of the antiviral immune surveillance.^[^ ^58^, ^59^ ^]^ TLRs share similar domain structures, including an ectodomain comprising leucine‐rich repeats that recognize PAMPs, a transmembrane domain, and a C‐terminal cytoplasmic domain, known as Toll‐interleukin‐1 receptor (IL‐1R) homology (TIR) domain. The TIR domain in the cytoplasm is crucial for signal transduction.^[^ ^3^, ^60^ ^]^

**Figure 3:**
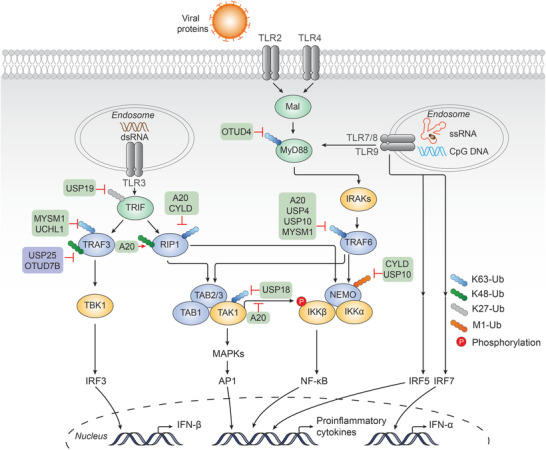
DUBs in TLR mediated signaling cascades. TLR2/4 and TLR7/8/9 recruit MyD88 and IRAKs, forming the MyD88 signaling complex. TRAF6 is subsequently recruited, synthesizing K63‐linked polyubiquitin chains that act as a scaffold for recruiting TAK1 and IKK complexes. TAK1 phosphorylates IKKbeta and activates IKK complex. TAK1 also activates the MAPK kinase family. Activation of IKK and MAPKs leads to the activation of transcription factors NF‐kappaB and AP1, respectively, and induces proinflammatory cytokines. TLR7/8/9 can initiate the activation of IRF5 and IRF7, whereas TLR3 recruits TRIF. Activated TRIF recruits both TRAF3 and RIP1. TRAF3 is crucial for recruiting the TBK1 complex following K63‐linked polyubiquitination. TBK1 recruits and phosphorylates IRF3 and promotes IFN‐beta production. DUBs that upregulate or downregulate the signaling are shown with purple or green back color, respectively. A detailed list of the target proteins and DUBs is included in Table 1.

TLRs initiate downstream signaling cascades by recruiting their respective adaptor proteins upon ligand binding (**Figure** **3**). Apart from TLR3, which recruits TIR‐domain‐containing adaptor‐inducing interferon‐*β* (TRIF), all TLRs involved in antiviral immunity recruit myeloid differentiation primary response 88 (MyD88).^[^ ^61^, ^62^ ^]^

TLR2/4, as well as TLR7/8/9 activation, induces the recruitment of MyD88 and the assembly of the MyD88 signaling complex, termed Myddosome, which consists of MyD88 itself and the IL‐1 receptor‐associated kinase (IRAK) family kinases IRAK4 and IRAK1/2.^[^ ^63^, ^64^ ^]^ TRAF6, cIAP1, and cIAP2 can also be recruited to Myddosome, functioning as ubiquitin ligases that mediate K48‐linked polyubiquitination of the tumor necrosis factor receptor‐associated factor 3 (TRAF3) and consequent degradation by the proteasome.^[^ ^65^ ^]^ In response to stimuli, Myddosome triggers IRAK4 autophosphorylation and activation, followed by IRAK4‐mediated phosphorylation and activation of IRAK1/2.^[^ ^63^, ^66^ ^]^ IRAKs are then dissociated from MyD88, resulting in the activation of TRAF6. TRAF6 forms a complex with two E2 enzymes, Ubc13 and Uev1A, which promotes the synthesis of K63‐linked polyubiquitin chains, thereby acting as a scaffold to recruit the downstream signaling molecules, TGF‐*β*‐activated kinase 1 (TAK1) and *κ*B kinase (IKK) complexes, through their ubiquitin‐binding subunits, TGF‐*β*‐activated kinase 1 and MAP3K7‐binding protein 2/3 (TAB2/3) and NEMO, respectively.^[^ ^67^ ^]^ As a homologous binding partner of both IKK*α*/IKK*β* complex and TANK‐binding kinase 1 (TBK1)/IKK*ε* complex, NEMO can be catalyzed with K27‐, K29‐, and M1‐linked ubiquitin chains, thereby regulating the phosphorylation of NF‐*κ*B and interferon regulatory factor 3 (IRF3) and subsequent translocation into the nucleus.^[^ ^68^, ^69^, ^70^ ^]^ Notably, linear ubiquitin chain assembly complex (LUBAC), which consists of heme‐oxidized IRP2 ubiquitin ligase 1L (HOIL‐1L), HOIL‐1‐interacting protein (HOIP), and Sharpin, catalyzes the M1‐linked ubiquitination (also known as linear ubiquitination) of NEMO, which is a prerequisite for recruiting the IKK complex (IKK*α* and IKK*β*). TAK1 not only induces the phosphorylation of IKK*β*, leading to the activation of IKK, but also activates the mitogen‐activated protein kinase (MAPK) kinase family. These kinases lead to the activation of transcription factors NF‐*κ*B and activator protein 1 (AP1) and induction of proinflammatory cytokines.^[^ ^71^, ^72^ ^]^ Of note, in the TLR7/8/9‐mediated signaling pathway, IRF7 can be directly recruited by both MyD88 and IRAK1, followed by IRAK1‐mediated phosphorylation and activation.^[^ ^73^ ^]^ TRAF6, TRAF3, and IKK*α* are proved to be necessary for this activation.^[^ ^74^, ^75^, ^76^ ^]^ Besides, IRF5 downstream of MyD88 and TRAF6 is indispensable for TLR7/8/9‐mediated production of proinflammatory cytokines.^[^ ^77^ ^]^

In contrast, TLR3 activation leads to the recruitment and subsequent activation of TRIF through TIR–TIR interaction.^[^ ^78^ ^]^ Activated TRIF binds TRAF3, a ubiquitin ligase crucial for the recruitment of TBK1 and the inhibitor of the IKK*ε* complex, following modification of TRAF3 with K63‐linked polyubiquitin.^[^ ^74^, ^75^ ^]^ The TBK1/IKK*ε* complex recruits and phosphorylates IRF3. The activated IRF3 dimerizes and enters the nucleus and induces type I IFN production. Additionally, TRIF also activates the NF‐*κ*B pathway. TRIF recruits and binds to receptor‐interacting serine/threonine‐protein kinase 1 (RIPK1, also known as RIP1), which activates TAK1. The activation of NF‐*κ*B is then mediated by a similar mechanism downstream of TRAF6. Therefore, TAK1 results in a variety of signaling cascades and ultimately initiate the transcription of proinflammatory cytokines.

Ubiquitination plays a critical role in TLR‐triggered MyD88 and TRIF signaling. Multiple E3 ligases have been identified to negatively regulate TLR signaling through K48‐linked polyubiquitination. Notably, Triad3A has been reported to target not only certain TLRs, including TLR3, 4, 5, and TLR9 but also RIP1 and TRAF3, ultimately leading to their degradation.^[^ ^79^, ^80^, ^81^ ^]^ The E3 ubiquitin ligase Parkin negatively regulates the antiviral signaling pathway by targeting TRAF3 for degradation; however, the process is inhibited by the mitochondrial protein PTEN‐induced kinase 1 (PINK1), which associates with TRAF3 via the kinase domain.^[^ ^82^, ^83^ ^]^ Smurf1/2 interacts with MyD88, leading to its K48‐linked ubiquitination and degradation, thus limiting the inflammatory response.^[^ ^84^ ^]^ Neuregulin receptor degradation protein 1 (Nrdp1) is another E3 ubiquitin ligase that leads to MyD88 degradation through K48‐linked ubiquitination.^[^ ^85^ ^]^ Intriguingly, Nrdp1 can also conjugate K63‐linked polyubiquitin on TBK1, thereby causing effects that are opposite. Similarly, TRIF can be targeted for K48‐linked polyubiquitination and subsequent degradation by the E3 ligase WW domain‐containing protein 2 (WWP2)^[^ ^86^ ^]^ and tripartite motif‐containing 38 (TRIM38).^[^ ^87^ ^]^ BICP0, a viral E3 ubiquitin ligase encoded by bovine herpes virus‐1,^[^ ^88^ ^]^ is another negative regulator that promotes the K48‐linked ubiquitination and degradation of TRAF6.^[^ ^89^ ^]^ TRAF6 itself is the E3 ligase that can target IRF7 for K63‐linked polyubiquitination. This process is negatively regulated by TARBP2 by its participation in the interaction between IRF7 and TRAF6.^[^ ^90^ ^]^

Deubiquitination precisely controls the abundance and activity of signaling molecules in TLR signaling, thereby avoiding dysregulation (Figure 3). Over the years, DUBs that participate in this pathway have been well characterized, A20 being the most widely studied. A20 is a potent inhibitor of TLR‐mediated signaling. It directly removes K63‐linked polyubiquitin chains from the signaling molecule TRAF6, which causes the termination of TLR‐induced activity.^[^ ^91^ ^]^ Nevertheless, the role of A20 is not that simple due to its structural features, including the presence of an OTU domain as well as seven zinc‐finger (ZnF) motifs, which confer both DUB activity and E3 ubiquitin ligase activity on A20, respectively. Whereas A20 removes K63‐linked polyubiquitin chains from its substrates, such as RIPK1, A20 also introduces K48‐linked polyubiquitin chains in the same substrate, thereby tagging it for proteasomal degradation.^[^ ^92^ ^]^ Additionally, A20 can bind polyubiquitin chains through its zinc‐finger motif, which leads to its specific binding to ubiquitinated NEMO. This allows phosphorylation of IKK to be sufficiently blocked by its upstream kinase TAK1, which inhibits NF‐*κ*B activation.^[^ ^93^ ^]^ Another study has shown that A20, with the help of the regulatory molecule Tax1 Binding Protein 1 (TAX1BP1), interacts with E2 enzymes Ubc13 and UbcH5c and triggers their ubiquitination and subsequent degradation, which antagonizes their interactions with the E3 ligases TRAF6, TRAF2, and cIAP1. The activities of these E3 ligases are consequently dampened.^[^ ^94^ ^]^ These findings together reveal the diverse and intricate roles of A20 in the inhibition of TLR signaling pathways. CYLD is another well‐studied DUB. In TLR‐mediated signaling, CYLD negatively regulates NF‐*κ*B signaling by cleaving K63‐linked polyubiquitin chains and M1‐linked polyubiquitin chains from RIPK1, TRAF2, and NEMO.^[^ ^95^, ^96^, ^97^ ^]^

Several DUBs in TLR signaling perform nondegradative roles. For example, USP19 was identified to play a role in downregulating TLR3/4‐mediated signaling. USP19 interacts with TRIF and removes the K27‐linked polyubiquitin moieties of TRIF, which inhibits the recruitment of TRIF to TLR3/4. In vitro and in vivo assays further demonstrated that USP19 negatively regulates TLR3/4‐mediated innate immune and inflammatory responses.^[^ ^98^ ^]^ OTUD4 was previously shown as a K48‐specific DUB that is important for maintaining the stability of alkylation repair enzymes.^[^ ^99^ ^]^ In another study, OTUD4 has been reported to harbor K63‐specific DUB activity following phosphorylation at Serine202 and Serine204. Furthermore, this activity requires an adjacent ubiquitin‐interacting motif that increases the affinity of OTUD4 for K63‐linked chains. OTUD4 can target MyD88, thereby negatively regulating the TLR‐mediated NF‐*κ*B pathway. Indeed, OTUD4‐deficient mice have been shown to exhibit increased inflammatory signaling in response to TLR stimulation.^[^ ^100^ ^]^ TRAF6 and TRAF3 are essential for mediating downstream signaling, and their activities are tightly regulated by several DUBs. Myb‐like, SWIRM, and MPN domains 1 (MYSM1) targets both TRAF3 and TRAF6 for K63‐linked deubiquitination and subsequent inactivation, subsequently terminating TLR‐mediated pathways for antiviral responses, which is evidenced genetically. Specifically, the SWIRM and metalloproteinase domains have been identified as responsible for the interaction with both TRAF3 and TRAF6, and the removal of K63‐linked polyubiquitin chains, respectively.^[^ ^101^ ^]^ USP4 has been identified as a potent negative regulator of TLR/IL‐1R signaling. USP4 interacts with and deubiquitinates TRAF6, thereby preventing the activation of NF‐*κ*B and AP‐1 transcription factors and subsequent immune responses. Moreover, *usp4*‐depleted zebrafish (*Danio rerio*) larvae have been shown to produce more proinflammatory cytokines and were more susceptible to endotoxic challenge following LPS treatment.^[^ ^102^ ^]^ Ubiquitin carboxyl‐terminal hydrolase L1 (UCHL1) cleaves K63‐linked polyubiquitin chains on TRAF3, which ultimately leads to reduced production of type I IFN, proinflammatory cytokines, and chemokines in response to high‐risk human papillomavirus (hrHPV) infection.^[^ ^103^ ^]^ TRAF family member‐associated NF‐*κ*B activator (TANK) interacts with both monocyte chemotactic protein‐1‐induced protein‐1 (MCPIP1) and USP10, leading to the cleavage of ubiquitin chains on TRAF6 and termination of NF‐*κ*B activation in response to TLR activation.^[^ ^104^ ^]^ Of note, USP10 also interacts with NEMO via MCPIP1 and leads to the removal of NEMO‐attached M1‐linked polyubiquitin chains, thus inhibiting the genotoxic NF‐*κ*B signaling cascade.^[^ ^40^ ^]^ In addition to interacting with USP10, MCPIP1 itself regulates cellular inflammation pathways with distinct mechanisms. A study showed that MCPIP1 negatively regulates c‐Jun N‐terminal kinase (JNK) and NF‐*κ*B activities, induced by LPS, IL‐1*β*, and tumor necrosis factor‐*α* (TNF‐*α*), by removing ubiquitin moieties from TRAF2, TRAF3, and TRAF6. Consistently, the fatal inflammatory syndrome was observed in MCPIP1‐deficient mice.^[^ ^105^ ^]^ In addition to its DUB activity, MCPIP1 restricts cellular inflammation via its RNase activity. Interestingly, several studies have shown that MCPIP1 RNase activity renders specific mRNA degradation of IL‐6, IL‐1*β*, and IL‐2.^[^ ^106^, ^107^ ^]^ Conversely, MCPIP1 can also be a positive regulator of antiviral immunity. MCPIP1 ribonuclease exhibits broad‐spectrum antiviral effects via the binding and degradation of viral RNA.^[^ ^108^ ^]^ Studies have found that MCPIP1 not only inhibits RNA viruses, such as Japanese encephalitis (JEV), dengue virus (DEN), sindbis virus, encephalomyocarditis, influenza A, hepatitis C virus (HCV), and human immunodeficiency virus (HIV), but also DNA viruses, such as the hepatitis B virus (HBV).^[^ ^108^, ^109^, ^110^, ^111^, ^112^, ^113^ ^]^ Moreover, while MCPIP1 can also promote IFN‐I‐mediated antiviral efficacy in a manner independent of its RNase and DUB activity, the underlying mechanism is not clear.^[^ ^114^ ^]^ Similarly, USP18 acts as a negative regulator of IFN responses by counteracting ISG15 conjugation upon viral infection.^[^ ^115^ ^]^ Additionally, it has been reported that USP18 also regulates TLR‐induced NF‐*κ*B activation by removing K63‐linked polyubiquitin chains of TAK1 and NEMO.^[^ ^116^ ^]^

It has been shown that some DUBs are related to the stability of signaling molecules in the TLR pathway. In response to LPS, USP25 prevents the K48‐linked ubiquitination of TRAF3 that is mediated by cIAP2. Deficiency in USP25 accelerates its degradation following TLR4 activation. Indeed, USP25 knockout mice exhibited increased susceptibility to LPS‐induced septic shock.^[^ ^117^ ^]^ In response to noncanonical NF‐*κ*B stimuli, OTUD7B displays K48‐specific DUB function, which inhibits TRAF3 proteolysis and prevents aberrant non‐canonical NF‐*κ*B activation through binding and deubiquitination of TRAF3.^[^ ^118^ ^]^

Although a large number of host DUBs that control TLR signaling have been characterized over the years, much of the data attempting to elucidate whether DUBs function in the context of defending viral infections or other pathogen infections are inconclusive. Hence, in this Review, we present a summary of the host DUBs that target key molecules in the TLR signaling pathway. It should be noted that the specific function of the host DUBs differs, depending on the PAMPs, cell type, and infection model examined. Further studies are needed to confirm the antiviral function of DUBs in TLR signaling.

### 3.2 DUBs in RLR Signaling

While TLR‐mediated signaling exerts extensive antiviral immune responses, this system has its limitations due to a lack of TLR expression in most nonimmune cells. Therefore, alternative PRRs, such as RLRs, are needed. RLRs are cytosolic RNA sensors that consist of three members: RIG‐I,^[^ ^119^ ^]^ melanoma differentiation‐associated protein 5 (MDA5),^[^ ^120^ ^]^ and laboratory of genetics and physiology 2 (LGP2),^[^ ^121^ ^]^ all of which are distributed in the cytoplasm. All RLRs contain a C‐terminal domain (CTD) and an intermediate RNA helicase domain. At the N‐termini, all RLRs, except for LGP2, possess tandem caspase recruitment domains (CARDs), which are necessary for downstream signal transduction.^[^ ^122^ ^]^ Although sharing similar domain architectures, they each have different ligand preferences: RIG‐I recognizes ssRNAs bearing a 5′‐triphosphate moiety, as well as short dsRNA (∼1kb) bearing 5′‐triphosphate or with a lower affinity 5′‐diphosphate, whereas MDA5 prefers to sense longer dsRNA.^[^ ^123^, ^124^, ^125^, ^126^, ^127^, ^128^ ^]^ LGP2 has been shown to engage in the recognition of and interaction between RNA and RIG‐I/MDA5, thus exerting a regulatory role in downstream signaling.^[^ ^129^, ^130^, ^131^ ^]^

**Figure 4:**
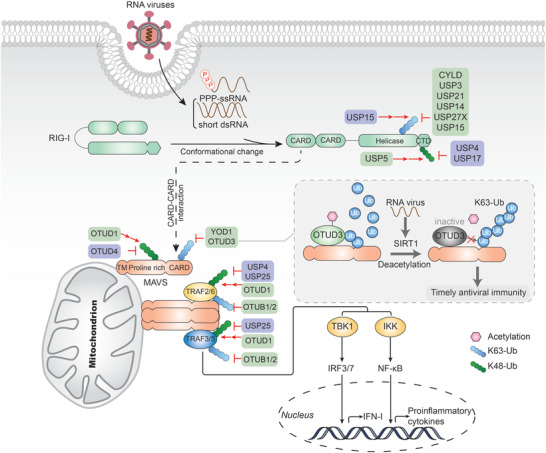
DUBs in RLR‐mediated signaling cascades. RIG‐I recognizes and binds to ssRNA bearing a 5’‐triphosphate moiety, as well as short dsRNA, leading to a conformational change that exposes CARDs. RIG‐I then binds MAVS through CARD–CARD interaction. MAVS oligomerizes and activates IRF3/7‐ and NF‐kappaB‐mediated signaling by recruiting TRAFs, leading to the production of type I IFN and proinflammatory cytokines. DUBs that upregulate or downregulate the signaling are shown with purple or green back color, respectively. A detailed list of the target proteins and DUBs is included in Table 1.

Among these three cytosolic RNA sensors, RIG‐I and MDA5 recognize and bind to microbial RNA through CTD, leading to a conformational change that exposes CARDs. This allows RIG‐I and MDA5 to interact with mitochondrial antiviral‐signaling protein (MAVS, also known as IPS‐1, VISA, and CARDIF) through CARD–CARD interaction.^[^ ^132^, ^133^ ^]^ MAVS further activates TBK1 and IKK by recruiting TRAF proteins. TBK1 and IKK complexes activate and phosphorylate IRF3 and NF‐*κ*B, respectively, leading to the induction of interferon and proinflammatory cytokines^[^ ^134^ ^]^ (**Figure** **4**).

Ubiquitination is also crucially involved in regulating RLR signaling. Structural remodeling of RIG‐I into active conformations occurs upon RNA binding, which allows RLRs undergoing oligomerization to be active. RIG‐I forms tetramers with the help of E3 ligase TRIM25, which can catalyze and form free K63‐linked polyubiquitin chains that bind to its CARDs.^[^ ^135^, ^136^ ^]^ Moreover, the E3 ligases TRIM25 and Riplet/RING finger protein 135 (RNF135) have been reported to conjugate K63‐linked polyubiquitin chains to RIG‐I CARDs, thus stabilizing this complex and boosting its activation.^[^ ^137^, ^138^, ^139^ ^]^ RIG‐I can also be conjugated with K63‐linked ubiquitin chains by E3 ligase TRIM4 and Mex‐3 RNA binding family member C (MEX3C), thus enhancing virus‐mediated type I IFN production.^[^ ^140^, ^141^ ^]^ Similarly, unanchored K63 polyubiquitin chains are indispensable in the oligomerization of MDA5, the resulting oligomer complex being stabilized by the covalent binding of polyubiquitin chains.^[^ ^135^, ^142^ ^]^ The E3 ligase TRIM31 interacts and catalyzes the K63‐linked polyubiquitination on MAVS, which promotes the formation of prion‐like aggregates of MAVS after viral infection.^[^ ^143^, ^144^ ^]^ Furthermore, the E3 ligase TRIM21 positively regulates innate antiviral immunity through K27‐linked polyubiquitination of MAVS.^[^ ^145^ ^]^ LUBAC negatively regulates RIG‐I and TRIM25‐mediated type I IFN induction by catalyzing M1‐linked ubiquitination and subsequent degradation of RIG‐I, as well as competing with TRIM25 for its association with RIG‐I, thus inhibiting RLR‐mediated signaling.^[^ ^146^ ^]^

K48‐linked polyubiquitination is needed for proteasomal degradation, thus negatively regulating the MAVS signaling cascade. The E3 ligases RNF125,^[^ ^147^ ^]^ RNF122,^[^ ^148^ ^]^ carboxyl terminus of Hsc70‐interacting protein (CHIP),^[^ ^149^ ^]^ and STIP1 homology and U‐box containing protein 1 (STUB1)^[^ ^150^, ^151^ ^]^ have been reported to attach K48‐linked ubiquitin chains on RIG‐I, and TRIM13^[^ ^152^ ^]^ works on MDA5. TRIM40 has been reported to interact with both RIG‐I and MDA5, catalyzing K27‐ and K48‐linked polyubiquitination, respectively, thereby promoting their degradation.^[^ ^153^ ^]^ A few E3 ligases, such as RNF5,^[^ ^152^ ^]^ SMAD specific E3 ubiquitin protein ligase 1(Smurf1),^[^ ^154^ ^]^ Smurf2,^[^ ^155^ ^]^ TAX1BP1,^[^ ^156^ ^]^ membrane‐associated RING finger (C3HC4) 5 (MARCH5),^[^ ^157^ ^]^ poly(rC)‐binding protein 1 (PCBP1),^[^ ^158^ ^]^ PCBP2,^[^ ^159^ ^]^ TRIM25,^[^ ^160^ ^]^ and receptor for activated C kinase 1 (RACK1),^[^ ^161^ ^]^ are responsible for the K48‐linked ubiquitination on MAVS.

Since K63‐linked ubiquitination of RIG‐I is crucial for its activation and signaling, it is not surprising that several DUBs are involved in downregulating RLR signaling to avoid overactivation (Figure 4). As the first DUB identified to deubiquitinate RIG‐I, Cylindromatosis (CYLD) removes its K63‐linked polyubiquitin chains, preventing any basal activation levels and thus dampening IFN production triggered by RIG‐I.^[^ ^162^ ^]^ Upon viral infection, or in the presence of a tumor necrosis factor, the CYLD expression level is downregulated to enhance IFN production. Notably, it has been shown that CYLD targets not only RIG‐I but also downstream TBK1, the kinase that phosphorylates and activates IRF3.^[^ ^162^ ^]^ In this case, Optineurin (Optn) was shown to dampen the response via targeting CYLD to TBK1 to impair its enzymatic activity.^[^ ^163^ ^]^ Similarly, USP3 also acts as a negative regulator of RIG‐I‐mediated signaling by removing K63‐linked ubiquitination on RIG‐I.^[^ ^164^ ^]^ Upon viral infection or ligand stimulation, USP3 binds to the CARD domain of RIG‐I and then removes its polyubiquitin chains through both the zinc‐finger Ub‐binding domain and USP catalytic domains. However, whether USP3 regulates RIG‐I in vivo remains unknown. Moreover, USP21 has been reported to remove K63‐linked ubiquitination of RIG‐I, resulting in impaired antiviral responses.^[^ ^165^ ^]^ Interestingly, USP21 presumably removes polyubiquitin chains from more than one lysine sites in either CARD or CTD domain, since USP21 can reverse the polyubiquitination mediated by both RNF135 and TRIM25. USP21‐deficient mice are more resistant to vesicular stomatitis virus (VSV) infection, concomitated with elevated IFN production.^[^ ^166^ ^]^ USP14, in another study, has also been shown to remove K63‐linked ubiquitin chains on RIG‐I. USP14‐specific inhibitor, IU1, was demonstrated to increase RIG‐I‐mediated type I IFN production and antiviral responses in vitro and in vivo.^[^ ^167^ ^]^ Recently, siRNA library screening identified USP27X as a negative regulator of type I IFN signaling by cleaving K63‐linked polyubiquitin chains from RIG‐I.^[^ ^168^ ^]^ While USP15 also plays a negative role in RIG‐I signaling, its activities are more difficult to decipher as it exhibits an opposite function in other contexts (see below).^[^ ^169^ ^]^

YOD1 (also known as OTUD2), a DUB of the OTU family, was the first identified DUB responsible for downregulating the K63‐linked polyubiquitination of MAVS. Following a Sendai virus infection, YOD1 was recruited to mitochondria and was shown to interact with MAVS. Subsequently, YOD1 removes the K63‐linked ubiquitin chain and abrogates the aggregation of MAVS, thus inhibiting not only IRF3 and p65 activation, but also IFN‐*β* production.^[^ ^170^ ^]^ It is worth mentioning that both YOD1 and TRIM31 interact with the proline‐rich domain of MAVS, indicating competition between these two enzymes with opposing roles. Recently, our previous research identified OTUD3 as an acetylation‐dependent DUB that directly deconjugates the K63‐linked polyubiquitin chains of MAVS and terminates downstream antiviral response.^[^ ^171^ ^]^ Intriguingly, the catalytic activity of OTUD3 is dependent on the acetylation of Lysine129. To confirm this, acetyl‐lysine is incorporated into a recombinant OTUD3 to generate a fully Lys129‐acetylated OTUD3 (OTUD3^Lys129‐Ac^) with chemical biology approaches. In vitro Ubiquitin‐AMC assay and deubiquitination assay demonstrated that OTUD3^Lys129‐Ac^ exhibits robust deubiquitinase activity toward K63‐linked ubiquitin chains and is more effective in targeting MAVS. Besides, this acetylated residue can be removed by sirtuin 1 (SIRT1) upon virus infection, which promptly inactivates OTUD3 and triggers the innate antiviral immune response promptly. Consistently, intensive antiviral immune response, diminished viral load, and morbidity were observed in OTUD3 knockout mice, and acetyl‐OTUD3 levels are negatively correlated with IFN‐*β* expression in influenza patients. These findings collectively establish OTUD3 as a new repressor of MAVS and unveil a novel regulatory mechanism through which the catalytic activity of OTUD3 is tightly tuned in the control of the timely antiviral defense.

Negative regulation of RLR signaling is also accomplished by targeting downstream molecules of MAVS. Coimmunoprecipitation analyses indicate that OTUB1 and OTUB2 associate with TRAF3 and TRAF6, negatively regulating antiviral responses by deubiquitinating their respective K63‐linked polyubiquitin chains.^[^ ^172^ ^]^ Because various components are involved in virus‐induced signaling, biochemical assays have demonstrated that OTUB1 and OTUB2 inhibit RIG‐I‐, MDA5‐, and MAVS‐mediated but not TBK1‐ and IRF3‐mediated signaling. Notably, the negative regulation of OTUB1 in TRAF3‐elicited antiviral innate immunity is enhanced by HSCARG (also known as NmrA‐like family domain containing 1, NMRAL1), which fosters the recruitment of OTUB1 to TRAF3 and abolishes its ubiquitination.^[^ ^173^ ^]^

Modulating the stability of key signaling molecules is another approach to inhibit RLR‐mediated signal transduction. A systematic functional screening revealed that USP5 can interact with and recruit STUB1 to mediate the degradation of RIG‐I, thus enhancing VSV replication.^[^ ^174^ ^]^ OTUD1 was also reported to inhibit IFN production during RNA virus infection. Mechanistic studies demonstrated that OTUD1 stabilizes Smurf1 by removing its ubiquitin chains, which allows the binding of Smurf1 to MAVS, TRAF3, and TRAF6 with subsequent degradation of each component of the MAVS/TRAF3/TRAF6 signalosome. Notably, OTUD1‐deficient mice were consistently shown to be more resistant to RNA virus infections when more antiviral cytokines were produced.^[^ ^175^ ^]^

Over the years, a growing number of DUBs have been identified to upregulate the RLR‐mediated signaling pathway. Since K48‐linked polyubiquitination may bring signaling molecules to proteasomal degradation, several DUBs were identified to reverse the process. Among them, USP17 and USP4 are the only two identified DUBs that are responsible for upregulating the stability of RIG‐I. USP17 facilitates RIG‐I‐ and MDA5‐mediated induction of type I IFN via the deubiquitination of RIG‐I, as well as MDA5. Furthermore, USP17‐deficiency inhibits the RNA‐virus‐induced expression of type I IFN and antiviral responses.^[^ ^176^ ^]^ USP4 interacts with and cleaves K48‐linked polyubiquitin chains from RIG‐I, thereby promoting RIG‐I‐mediated IFN‐*β* signaling and inhibiting VSV replication. Following virus‐induced RIG‐I activation, USP4 expression was shown to attenuate. Additionally, overexpression of USP4 dramatically enhanced the RIG‐I protein level, while knockdown of USP4 had an opposite effect.^[^ ^177^ ^]^ Although multiple E3 ligases responsible for the K48‐linked ubiquitination of MAVS have been identified over the past years, the corresponding DUBs remained elusive until the identification of OTUD4. It has been shown by DUB screening and biochemical analyses that OTUD4 removes K48‐linked polyubiquitin chains on MAVS in response to viral infection, thereby inhibiting the proteasomal degradation of MAVS and triggering downstream signaling.^[^ ^178^ ^]^ This is supported by the observation that knockout or knockdown of OTUD4 impairs the RNA virus‐triggered expression of type I interferon and enhances VSV replication, both in vitro and in vivo.^[^ ^178^ ^]^ Interestingly, as mentioned before, OTUD4 cleaves K63‐linked polyubiquitin chains after it is phosphorylated.^[^ ^100^ ^]^ Therefore, it might be the in vivo protein modification that strongly altered the chain specificity of OTUD4. TRAF6 and TRAF3 play crucial roles in the RLR‐mediated antiviral signaling pathway by recruiting other related proteins to form a complex that facilitate NF‐*κ*B signaling. A study has demonstrated that USP4 is capable of targeting TRAF6 for K48‐linked deubiquitination and inhibiting enterovirus 71 (EV71) replication.^[^ ^179^ ^]^ USP25 interacts with TRAF3 and TRAF6 following RNA virus or DNA virus infection and prevents TRAF3 and TRAF6 from proteasomal degradation via deubiquitinating K48‐linked polyubiquitin chains. Furthermore, USP25 deficiency results in enhanced ubiquitination and turnover of TRAF6 and TRAF3. Consistently, USP25 deficient mice were shown to be more susceptible to H5N1 or HSV‐1 infection compared to their wild‐type counterparts.^[^ ^180^ ^]^

In addition to preventing signaling proteins from degradation by removing K48‐linked polyubiquitin chains, USP15 was shown to upregulate RLR signal transduction by indirectly attaching K63‐linked polyubiquitin chains on RIG‐I. As mentioned previously, the E3 ligase TRIM25 is responsible for K63‐linked polyubiquitination of RIG‐I and it can be degraded by LUBAC‐mediated polyubiquitination. A study has demonstrated that USP15 deubiquitinates TRIM25, thus counteracting the LUBAC‐dependent degradation of TRIM25.^[^ ^181^ ^]^ Mechanistically, USP15 interacts with TRIM25 during the late stage of a viral infection, removes the LUBAC‐induced K48‐linked polyubiquitination of TRIM25 at its SPRY domain, and ultimately leads to sustained type I IFN expression. Combined with the negative role of USP15 mentioned above, the opposing roles suggest that USP15 exerts disparate effects depending on substrate recognition or infection severity, thereby underscoring the tight regulation of cellular immune responses.

### 3.3 DUBs in STING Signaling

**Figure 5:**
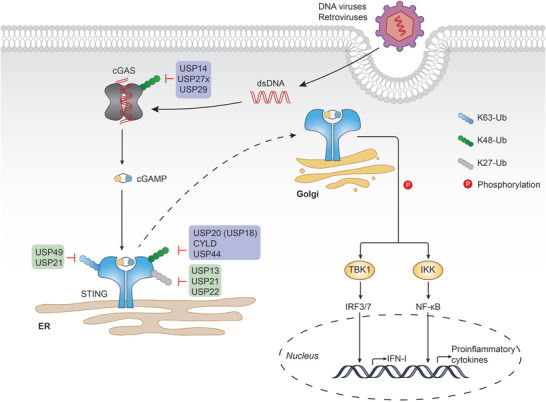
DUBs in cGAS‐STING mediated signaling cascades. cGAS recognizes DNA in the cytosol and is activated upon direct binding, which catalyzes the formation of cGAMP. cGAMP then binds to and activates STING. STING then translocates from the ER to the Golgi, where it interacts with and phosphorylates TBK1 and IKK. IRF3/7 and NF‐kappaB are activated by TBK1 and IKK, respectively, leading to the production of type I IFN and proinflammatory cytokines. DUBs that upregulate or downregulate the signaling are shown with purple or green back color, respectively. A detailed list of the target proteins and DUBs is included in Table 1.

Almost all microbial pathogens rely on DNA for their replication. Normally, host DNA resides in the nucleus and mitochondria. Upon infection, pathogen DNA is exposed in the cytosol. Thus, sensing DNA in the cytosol is a crucial innate immune strategy against both bacterial and viral pathogens.^[^ ^182^ ^]^ The main pathway that mediates the immune response to DNA is governed by cGAS.^[^ ^183^ ^]^ cGAS recognizes DNA and is activated through direct binding, which catalyzes the formation of cGAMP.^[^ ^184^, ^185^, ^186^ ^]^ cGAMP then binds to and activates the stimulator of interferon genes (STING, also known as MITA,^[^ ^187^ ^]^ ERIS,^[^ ^188^ ^]^ or MPYS^[^ ^189^ ^]^), an endoplasmic reticulum (ER)‐localized adaptor.^[^ ^190^, ^191^ ^]^ Upon activation, STING translocates to the Golgi and interacts with TBK1 and IKK, which phosphorylates IRF3 and NF‐*κ*B inhibitor I*κ*B*α*, respectively.^[^ ^191^, ^192^, ^193^ ^]^ The TBK1/IKK*ε* and IKK pathways are subsequently activated, thus leading to the induction of type I interferons and proinflammatory cytokines^[^ ^194^, ^195^, ^196^ ^]^ (**Figure** **5**).

Ubiquitination is equally important in STING signaling. TRIM56, an interferon‐induced E3 ubiquitin ligase, interacts with STING and targets it for K63‐linked ubiquitination, which induces STING dimerization and recruitment of TBK1.^[^ ^197^ ^]^ Likewise, the E3 ligase TRIM32 targets STING for K63‐linked ubiquitination and shares the same effects with TRIM56.^[^ ^198^ ^]^ Autocrine motility factor receptor (AMFR) catalyzes K27‐linked polyubiquitination of STING, which serves as an anchoring platform for recruiting TBK1 and promotes its translocation to the perinuclear microsomes.^[^ ^199^ ^]^ In addition to STING, cGAS can also be ubiquitinated to facilitate signaling. For example, the E3 ligase RNF185 attaches K27‐linked polyubiquitination to cGAS, thereby promoting its enzymatic activity.^[^ ^200^ ^]^ TRIM56 targets cGAS through monoubiquitination, which results in a remarkable increase in its dimerization, DNA‐binding activity, and cGAMP production.^[^ ^201^ ^]^ Another E3 ubiquitin ligase that mediates the monoubiquitination of cGAS is RING finger‐interacting protein with C kinase (RINCK, also known as TRIM41), which is critical for cGAS activation.^[^ ^202^ ^]^

The negative role of ubiquitination in the cGAS‐STING pathway is executed by the E3 ligases RNF5,^[^ ^203^ ^]^ TRIM29,^[^ ^204^ ^]^ and TRIM30,^[^ ^205^ ^]^ all of which target STING for K48‐linked ubiquitination and subsequent proteasomal degradation. Interestingly, the E3 ligase RNF26 competitively promoted K11‐linked polyubiquitination of STING at the same residue targeted by RNF5, thus protecting it from RNF5‐mediated degradation.^[^ ^206^ ^]^ Besides, K48‐linked ubiquitination of cGAS has been reported as a recognition signal for p62‐dependent selective autophagic degradation, although the E3 ubiquitin ligase(s) involved has not been determined yet.^[^ ^207^ ^]^

The K48 ubiquitin linkage type of cGAS is further supported by USP14, which can be recruited by the E3 ligase TRIM14 to cleave the K48‐linked ubiquitin chain of cGAS, thus promoting its stability (Figure 5). TRIM14 knockout mice are highly susceptible to lethal HSV‐1 infection due to impaired type I IFN production, which suggests a crucial role of USP14 in facilitating innate antiviral signaling.^[^ ^207^ ^]^ Apart from targeting RIG‐I, USP27x was previously identified as another DUB that regulates the stability of cGAS. Unlike USP14, USP27x directly interacts with cGAS and releases the K48‐linked polyubiquitin chains during viral infection.^[^ ^208^ ^]^ Recently, a study reported that USP29 interacts with cGAS, hydrolyzes K48‐linked polyubiquitin chains on cGAS, and stabilizes cGAS in uninfected cells, or after HSV‐1 stimulation.^[^ ^209^ ^]^ Following HSV‐1 infection, USP29 knockout mice were shown to be hypersensitive to HSV‐1 and produced less type I IFNs and proinflammatory cytokines than the wildtype control. Moreover, Trex1 deficiency resulted in the hyperproduction of type I IFNs and proinflammatory cytokines and have been associated with autoimmune disorders, such as Aicardi‐Goutieres syndrome (AGS) and systemic lupus erythematosus (SLE). Additionally, Trex1 knockout mice have been previously characterized to exhibit severe systemic inflammation and even lethal autoimmune responses.^[^ ^210^ ^]^ In the study, genetic ablation of USP29 in Trex1 knockout mice attenuated the related pathological symptoms, highlighting the crucial role of USP29 in the regulation of cGAS. Similar to cGAS, extensive studies have been performed on the discovery of DUBs involved in the cleavage of the K48‐linked ubiquitin chain on STING. For instance, USP20 was shown to interact with and remove K48‐linked ubiquitin chains from STING after HSV‐1 infection, thereby stabilizing STING and promoting cellular antiviral responses.^[^ ^211^ ^]^ Although deubiquitinating STING alone, USP20 can be recruited by USP18, thus causing USP20 to remove K48‐linked polyubiquitin chains more efficiently, even at a low dose. Furthermore, a deficiency of either USP20 or USP18 has been reported to affect the stability of STING, and subsequent production of type I IFN, upon viral infection. Notably, this process is independent of the enzymatic activity of USP18.^[^ ^212^ ^]^ CYLD is another DUB that removes the K48‐linked polyubiquitin chains from STING at K150. CYLD knockout mice were shown to be more susceptible to HSV‐1 infection than their wild‐type littermates.^[^ ^213^ ^]^ More recently, a study identified USP44 as a novel positive regulator of STING.^[^ ^214^ ^]^ Upon DNA virus infection, USP44 was recruited to the membrane‐localized STING and selectively removed its K48‐linked polyubiquitin moieties at K236. Additionally, USP44‐deficient mice were more susceptible following HSV‐1 infection. All three DUBs seem to prevent STING from proteasome‐dependent degradation through uncoupling K48‐linked ubiquitination after DNA virus infection. However, compared to USP20 and USP44, CYLD‐mediated deubiquitination lacks target specificity in the control of innate immune signaling, as it also targets TRAF2 and RIG‐I.^[^ ^162^, ^215^ ^]^ USP20 seems more complicated, as it has also been shown to deubiquitinate and stabilize Unc‐51 like autophagy activating kinase 1 (ULK1), which is a negative regulator of STING.^[^ ^216^ ^]^ Besides, reconstitution experiments demonstrated that USP44‐mediated regulation of STING signaling is independent of USP20 and CYLD.^[^ ^214^ ^]^ In light of these, it is likely that USP20, CYLD, and USP44 are not functionally redundant. They may target STING spatially and temporally to fine‐tune the onset and termination of innate immune responses against DNA viruses.

DUBs also play a role in downregulating the STING pathway (Figure 5). K63‐linked ubiquitination of STING can be released by USP49 after HSV‐1 stimulation. This leads to the inhibited aggregation of STING and the recruitment of TBK1, finally impairing antiviral responses. USP49 knockout mice were shown to exhibit resistance to lethal HSV‐1 infection, along with decreased HSV‐1 replication.^[^ ^217^ ^]^ DUBs can also cleave atypical polyubiquitin chains on STING, as demonstrated by USP13 deconjugating the K27/33‐linked polyubiquitin chains from STING, which impairs the recruitment of TBK1 to STING. In line with this, USP13‐deficient mice restricted HSV‐1 replication in vivo and were more resistant to HSV‐1 infection.^[^ ^218^ ^]^ USP21 is another DUB that hydrolyzed the K27/K63‐linked polyubiquitin chain on STING following p38‐mediated phosphorylation, thus negatively regulating the production of type I interferons induced by the DNA virus.^[^ ^219^ ^]^ STING signaling can also be negatively regulated by USP22, which interacts with USP13 and specifically cleaves K27‐linked ubiquitin chains on STING, while the catalytic activity of USP22 is dispensable for this effect.^[^ ^174^ ^]^

In light of these studies, it is conceivable that STING is regulated by numerous DUBs in a spatial and temporal manner so as to initiate and terminate innate antiviral responses more accurately against non‐self. However, it remains to be investigated how these ubiquitination and deubiquitination processes are spatially and temporally coordinated.

## 4 DUBs in Downstream Signaling Molecules of PRRs

**Figure 6:**
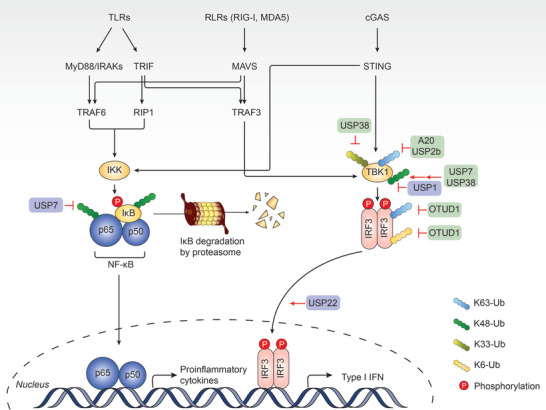
DUBs in key signaling molecules downstream of PRRs. TLR‐, RLR‐, and cGAS‐STING‐mediated signaling pathways share common signaling molecules such as IKKs, TBK1, and their respective substrates, NF‐kappaB, and IRF3. IKK phosphorylates IkappaB, leading to its ubiquitination and degradation. Thus, NF‐kappaB is free to enter the nucleus, promoting the production of proinflammatory cytokines. TBK1 phosphorylates and activates IRF3. Afterwards, IRFs dimerizes and translocates to the nucleus, leading to the production of type I IFN. DUBs that upregulate or downregulate the signaling are shown with purple or green back color, respectively. A detailed list of the target proteins and DUBs is included in Table 1.

Although different sensors signal through distinct adaptor proteins, the antiviral immune pathways converge on common signaling molecules downstream of these adaptors. Among these common molecules are IKKs, TBK1, as well as their respective substrates, and transcription factors NF‐*κ*B and IRF3. IKK phosphorylates I*κ*B, the inhibitory subunit of NF‐*κ*B that sequesters NF‐*κ*B in the cytoplasm, and the phosphorylated I*κ*B leads to its own ubiquitination and subsequent proteasomal degradation, thus enabling the release of NF‐*κ*B to the nucleus and the transcription of proinflammatory cytokines.^[^ ^220^ ^]^ Conversely, TBK1, along with its homolog IKK*ε*, mediates phosphorylation and activation of IRF3.^[^ ^196^, ^221^, ^222^ ^]^ Upon activation, IRF3 dimerizes and translocates to the nucleus, thus leading to the production of type I IFN^[^ ^194^ ^]^ (**Figure** **6**).

Since ubiquitination of I*κ*B is indispensable for NF‐*κ*B activation, DUBs are equally crucial (Figure 6). Notably, USP7 has been shown to play dual roles in the regulation of NF‐*κ*B. A study reported that USP7 is critical for target gene transcription, whereby USP7 targeted NF‐*κ*B p65 for K48‐linked deubiquitination, increased the protein stability of p65, and ultimately led to increased transcription. Specifically, after receptor activation, USP7 is recruited to NF‐*κ*B target promoters and interacts with NF‐*κ*B in a DNA‐binding‐dependent manner.^[^ ^223^ ^]^ Meanwhile, USP7 can also exert the opposing effect by deubiquitinating NEMO and consequently reducing degradation of I*κ*B*α* in response to TNF‐*α*, which in turn retains NF‐*κ*B in the cytoplasm and inhibits NF‐*κ*B activity.^[^ ^224^ ^]^ The dual role reveals that USP7 is not particularly specific, and its function differs in different cellular localizations. Moreover, A20 is an enzyme with both deubiquitinating and ubiquitin ligating activities. It has been reported that A20 inhibits both the degradation and processing of p105, the precursor of p50 upstream to IKK activation by affecting p105 ubiquitination.^[^ ^225^ ^]^

Optimal activation of TBK1 is critical for the initiation of antiviral innate immunity and the maintenance of immune homeostasis. TBK1 can be ubiquitinated with K63‐linked polyubiquitin chains by the E3 ligase RNF128, leading to its activation and ultimate production of type I IFN.^[^ ^226^ ^]^ In contrast, the E3 ligases Triad3A, protein deltex‐4 (DTX4), and TRAF‐interacting protein (TRIP) conjugate K48‐linked polyubiquitin chains on TRAF3 and TBK1, resulting in their proteasomal degradation and inhibition of type I IFN production.^[^ ^80^, ^227^, ^228^ ^]^ DUBs targeting TBK1 cannot be ignored as several DUBs have been shown to be involved in the regulation of TBK1 activity (Figure 6). A20 has been identified as a negative regulator of IRF3 signaling.^[^ ^229^, ^230^, ^231^ ^]^ A study provided an underlying mechanism whereby A20, together with TAX1BP1, disrupts K63‐linked polyubiquitination of TBK1, thus restricting antiviral signaling.^[^ ^232^ ^]^ Similarly, USP2b was demonstrated to remove K63‐linked polyubiquitin chains from TBK1 to dampen TBK1 kinase activity and terminate TBK1 activation, thus negatively regulating IFN‐*β* signaling and the antiviral immune response.^[^ ^233^ ^]^ Of note, the interaction between USP2b and TBK1 is promoted during viral infection, suggesting USP2b as negative feedback to avoid excessive antiviral responses. USP7, in another study, was found to inhibit type I IFN signaling by indirectly targeting TBK1. Specifically, TRIM27 is a well‐characterized E3 ubiquitin ligase that negatively regulates antiviral signaling pathways by targeting TBK1 for ubiquitination and subsequent degradation.^[^ ^234^ ^]^ USP7 interacts with TRIM27 and promotes the stability of TRIM27. This USP7‐TRIM27 axis allows negative modulation of antiviral type I IFN signaling.^[^ ^235^ ^]^

To date, USP38 and USP1 are the only two DUBs that have been identified to regulate the stability of TBK1 (Figure 6). USP38 interacts with the active form of TBK1 via NLR family pyrin domain containing 4 (NLRP4) and then removes K33‐linked ubiquitin chains from TBK1 at Lys670, which allows DTX4 and TRIP to catalyze K48‐linked ubiquitination on the same residue. This contributes to the degradation of TBK1, thus negatively regulating type I IFN signaling.^[^ ^236^ ^]^ In contrast, USP1 forms a complex with UAF1 and enhances TLR3/4 and RIG‐I‐induced IRF3 activation and subsequent IFN‐*β* secretion by binding to TBK1 and removing its K48‐linked polyubiquitin chains, thus preventing it from proteasomal degradation.^[^ ^237^ ^]^ ML323, a specific inhibitor of USP1/UAF1 was found in the same study to enhance viral replication both in vitro and in vivo, thus adding evidence for the positive role of the USP1/UAF1 complex.^[^ ^237^ ^]^

As for the downstream molecule of TBK1, increasing evidence has shown the regulation of IRF3 activity via the ubiquitin system. Multiple E3 ubiquitin ligases, such as TRIM26, Pin1, RBCC protein interacting with PKC 1 (RBCK2), Ro52, and RTA‐associated ubiquitin ligase (RAUL, also known as KIAA10 or UBE3C) target IRF3 for K48‐linked polyubiquitination, thus negatively regulating the antiviral innate immune response.^[^ ^238^, ^239^, ^240^, ^241^, ^242^, ^243^ ^]^ To date, at least three DUBs have been reported to regulate interferon induction by modulating IRF3 activation and translocation (Figure 6). OTUD1 is a DUB that directly interacts with IRF3 and cleaves the K63‐linked polyubiquitin chains on IRF3 at the residue Lysine98. This leads to the inhibition of IRF3 nuclear translocation as well as its transcriptional activity.^[^ ^244^ ^]^ Moreover, a recent study reported OTUD1 also reverses K6‐linked ubiquitination from IRF3 upon viral infection. This ubiquitination is essential for the DNA binding capacity of IRF3. Hence, OTUD1 cleaves the K6‐linked ubiquitin chain and leads to the disassociation of IRF3 from the promoter region of target genes, thereby attenuating type I IFN induction. Following viral infection, OTUD1 knockout mice were shown to produce more type I IFNs and proinflammatory cytokines and were more resistant to lethal viral infection.^[^ ^245^ ^]^ Recently, cytoplasmic USP22 was found to promote nuclear translocation of IRF3. Mechanistically, upon viral stimulation, USP22 deubiquitinates and stabilized karyopherin subunit alpha 2 (KPNA2), a protein of the Importin *α* family that is involved in the import of proteins into the cell nucleus, thus efficiently boosting nuclear translocation of IRF3 and subsequent antiviral responses.^[^ ^246^ ^]^

## 5 Viral DUBs Targeting Key Molecules in Immune Response

Our discussion in the preceding sections of this review focused on how the host performs antiviral immune responses with DUBs. A list of host DUBs targeting key molecules in three major innate immune pathways is summarized in **Table** **1**. However, viruses have also evolved strategies to counteract host responses or evade host defenses. Similar to host DUBs, viral DUBs also target key signaling molecules in host innate immune pathways (**Table** **2**).

**Table 1:** Host DUBs targeting key molecules in innate immunity

Host targets	DUBs involved	Linkage type	Signaling function	Refs.
*Signaling inhibition*				
MyD88	OTUD4	K63	Inhibiting TLR/NF‐*κ*B signaling	^[^ ^100^ ^]^
TRIF	USP19	K27	Inhibiting TLR3/4‐mediated signaling	^[^ ^98^ ^]^
TRAF6	A20	K63	Terminating TLR‐induced activity	^[^ ^91^ ^]^
	USP4	K63	Preventing NF‐*κ*B and AP1 activation, inhibiting TLR/IL‐1R signaling	^[^ ^102^ ^]^
	USP 10	K63	Interacting with TANK and MCPIP1, terminating NF‐*κ*B activation in response to TLR	^[^ ^104^ ^]^
	MYSM1	K63	Terminating TLR‐mediated antiviral responses	^[^ ^101^ ^]^
	OTUD1	K48	Inhibiting IFN production	^[^ ^175^ ^]^
	OTUB1/2	K63	Inhibiting antiviral responses	^[^ ^172^ ^]^
TRAF3	MYSM1	K63	Terminating TLR‐mediated antiviral responses	^[^ ^101^ ^]^
	UCHL1	K63	Inhibiting IFN production	^[^ ^103^ ^]^
	OTUD1	K48	Inhibiting IFN production	^[^ ^175^ ^]^
	OTUB1/2	K63	Inhibiting antiviral responses	^[^ ^172^ ^]^
RIP1 (also known as RIPK1)	A20	K63	Inhibiting NF‐*κ*B signaling	^[^ ^92^ ^]^
	CYLD	K63	Inhibiting NF‐*κ*B signaling	^[^ ^95^ ^]^
	A20	K48	Inducing A20 degradation with E3 ligase activity, inhibiting NF‐*κ*B signaling	^[^ ^92^ ^]^
NEMO	A20	N/A	Binding to ubiquitinated NEMO, blocking IKK phosphorylation, inhibiting NF‐*κ*B activation	^[^ ^93^ ^]^
	CYLD	M1	Inhibiting NF‐*κ*B signaling	^[^ ^97^ ^]^
	USP10	M1	Interacting with NEMO via MCPIP1, inhibiting the genotoxic NF‐*κ*B signaling	^[^ ^40^ ^]^
TAK1	USP18	K63	Inhibiting TLR/NF‐*κ*B signaling	^[^ ^116^ ^]^
RIG‐I	USP5	K48	Interacting with STUB1, mediating RIG‐I degradation, enhancing VSV replication	^[^ ^174^ ^]^
	CYLD	K63	Inhibiting IFN production	^[^ ^162^ ^]^
	USP3	K63	Inhibiting antiviral responses upon viral infection	^[^ ^164^ ^]^
	USP21	K63	Inhibiting antiviral responses	^[^ ^165^ ^]^
	USP14	K63	Inhibiting antiviral responses	^[^ ^167^ ^]^
	USP27X	K63	Inhibiting antiviral responses	^[^ ^168^ ^]^
	USP15	K63	Inhibiting antiviral responses	^[^ ^169^ ^]^
MAVS	YOD1	K63	Inhibiting IRF3 and p65 activation, IFN‐*β* production, inhibiting antiviral responses	^[^ ^170^ ^]^
	OTUD1	K48	Inhibiting IFN production	^[^ ^175^ ^]^
	OTUD3	K63	Inhibiting antiviral responses	^[^ ^171^ ^]^
STING	USP49	K63	Inhibiting antiviral responses after HSV‐1 infection	^[^ ^217^ ^]^
	USP21	K63	Inhibiting type I interferon production induced by DNA virus	^[^ ^219^ ^]^
	USP13	K27/33	Impairing the recruitment of TBK1 to STING, inhibiting antiviral responses	^[^ ^218^ ^]^
	USP21	K27	Inhibiting type I interferon production induced by DNA virus	^[^ ^219^ ^]^
	USP22	K27	Interacting with USP13	^[^ ^174^ ^]^
TBK1	A20	K63	Inhibiting antiviral responses with TAX1BP1	^[^ ^232^ ^]^
	USP2b	K63	inhibiting TBK1 kinase activity, terminating TBK1 activation, inhibiting IFN‐*β* signaling	^[^ ^233^ ^]^
	USP7	K48	Interacting with and stabilizing TRIM27, inhibiting antiviral type I IFN signaling	^[^ ^235^ ^]^
	USP38	K33	Interacting with TBK1 via NLRP4, allowing DTX4‐ and TRIP‐ mediated K48‐linked polyubiquitination, promoting TBK1 degradation, inhibiting IFN‐I signaling	^[^ ^236^ ^]^
IRF3	OTUD1	K63	Inhibiting IRF3 nuclear translocation and its transcriptional activity	^[^ ^244^ ^]^
		K6	Disassociation of IRF3 from the promoter region of target genes, inhibiting type I IFN induction	^[^ ^245^ ^]^
*Signaling activation*				
TRAF3	USP25	K48	Preventing TRAF3 degradation following virus infection, facilitating antiviral responses	^[^ ^117^, ^180^ ^]^
	OTUD7B	K48	Inhibiting TRAF3 proteolysis, preventing aberrant non‐canonical NF‐*κ*B activation	^[^ ^118^ ^]^
RIG‐I	USP15	K63	Removing K48‐linked polyubiquitin chains on TRIM25, promoting IFN‐I expression	^[^ ^181^ ^]^
	USP4	K48	Stabilizing RIG‐I, prolonging IFN induction	^[^ ^177^ ^]^
	USP17	K48	Stabilizing RIG‐I, facilitating antiviral responses	^[^ ^176^ ^]^
MAVS	OTUD4	K48	Inhibiting MAVS degradation, triggering antiviral signaling	^[^ ^178^ ^]^
TRAF6	USP4	K48	Inhibiting TRAF6 degradation, inhibiting EV71 replication	^[^ ^179^ ^]^
	USP25	K48	Facilitating antiviral responses	^[^ ^180^ ^]^
cGAS	USP14	K48	Recruited by TRIM14, stabilizing cGAS, facilitating antiviral signaling	^[^ ^207^ ^]^
	USP27x	K48	Stabilizing cGAS, regulating DNA‐mediated signaling	^[^ ^208^ ^]^
	USP29	K48	Stabilizing cGAS, facilitating antiviral responses	^[^ ^209^ ^]^
STING	USP20 (USP18)	K48	Stabilizing STING, promoting antiviral responses after HSV‐1 infection, recruited by USP18 to increase efficiency	^[^ ^212^ ^]^
	CYLD	K48	Stabilizing STING, facilitating antiviral responses	^[^ ^213^ ^]^
	USP44	K48	Stabilizing STING, facilitating antiviral responses upon DNA virus infection	^[^ ^214^ ^]^
TBK1	USP1	K48	Preventing TBK1 degradation, enhancing TLR3/4‐ and RIG‐I‐induced IRF3 activation and IFN‐*β* secretion	^[^ ^237^ ^]^
p65	USP7	K48	Stabilizing p65, increasing 65 transcription	^[^ ^223^ ^]^
IRF3	USP22	K48	Stabilizing KPNA2, promoting IRF3 nuclear translocation, promoting antiviral responses	^[^ ^246^ ^]^

**Table 2:** Viral DUBs in host innate antiviral immunity

Virus	Viral DUB	Host targets	Refs.
**DNA virus**			
*Herpesvirus family*			
Herpes simplex virus 1 (HSV‐1)	UL36^USP^	TRAF3; I*κ*B*α*	^[^ ^247^, ^248^ ^]^
Kaposi's sarcoma‐associated herpesvirus (KSHV)	ORF64^USP^	RIG‐I	^[^ ^252^, ^254^ ^]^
Epstein‐Barr virus (EBV)	BPLF1	TRAF6; NEMO; I*κ*B*α*	^[^ ^257^, ^258^ ^]^
*Hepadnavirus family*			
Hepatitis B virus (HBV)	HBx	RIG‐I; TRAF3	^[^ ^255^, ^256^ ^]^
**RNA virus**			
*Coronavirus (CoV)*			
SARS‐CoV	SARS‐CoV PLP	RIG‐I, STING, TRAF3, TBK1, IRF3	^[^ ^261^, ^262^, ^263^ ^]^
MERS‐CoV	MERS‐CoV PLP	IRF3	^[^ ^259^, ^260^ ^]^
HCoV‐NL63	HCoV‐NL63 PLP2	RIG‐I, TBK1, IRF3, STING	^[^ ^269^ ^]^
Mouse hepatitis virus (MHV)	MHV PLP2	IRF3, TBK1	^[^ ^270^, ^271^ ^]^
Porcine epidemic diarrhea virus (PEDV)	PEDV PLP2	RIG‐I, STING	^[^ ^272^ ^]^
*Arterivirus family*			
Equine arteritis virus (EAV)	EAV PLP2	RIG‐I	^[^ ^273^ ^]^
Porcine reproductive and respiratory syndrome virus (PRRSV)	PRRSV PLP2	RIG‐I, I*κ*B*α*	^[^ ^273^, ^275^ ^]^
Simian hemorrhagic fever virus (SHFV)	SHFV PLP2	RIG‐I	^[^ ^273^ ^]^
Lactate dehydrogenase‐elevating virus (LDV)	LDV PLP2	RIG‐I	^[^ ^273^ ^]^
*Nairovirus family*			
Crimean‐Congo hemorrhagic fever virus (CCHFV)	CCHFV out	RIG‐I	^[^ ^273^ ^]^
*Picornavirus family*			
Foot‐and‐mouth disease virus (FMDV)	L^pro^	RIG‐I, TBK1, TRAF3, TRAF6	^[^ ^276^ ^]^
Seneca valley virus (SVV)	SVV 3C^pro^	RIG‐I, TBK1, TRAF3	^[^ ^277^ ^]^

Virus‐encoded DUBs have been identified in DNA viruses. An example of a DNA virus‐encoded DUB that interferes with the host antiviral immune responses is the herpes simplex virus 1 (HSV‐1) ubiquitin‐specific protease (UL36^USP^). Specifically, UL36^USP^ has been reported to act as a viral protein‐mediated immune antagonist by deubiquitinating TRAF3 and dampening IFN‐*β* signaling. The UL36^USP^ knockout virus leads to increased production of IFN‐*β* compared to wild‐type virus.^[^ ^247^ ^]^ UL36^USP^ was also shown to restrict NF‐*κ*B activation in the DNA sensing signaling pathway to evade host antiviral immunity. Mechanistically, UL36^USP^ cleaves polyubiquitin chains from I*κ*B*α* and abrogates proteasomal degradation of I*κ*B*α*, thus inhibiting NF‐*κ*B activation.^[^ ^248^ ^]^ In addition, UL36^USP^ dampens the IFN‐*β*‐induced activation of interferon‐sensitive response element (ISRE) promoter and the transcription of ISGs, by specifically binding to the interferon *α* and *β* receptor subunit 2 (IFNAR2) subunit, and blocks the association between Janus kinase 1 (JAK1) and IFNAR2. This study underscores the UL36^USP^‐IFNAR2 interaction as a novel evasion strategy exploited by HSV‐1.^[^ ^249^ ^]^ Homologs of UL36^USP^ have also been identified in other viruses in the Herpesviridae family, such as human cytomegalovirus (HCMV), murine *γ*herpes virus 68 (MHV‐68), Marek's disease virus (MDV), and Kaposi's sarcoma virus (KSHV).^[^ ^250^, ^251^, ^252^, ^253^ ^]^ Although the DUB activity was identified, the role of these viral DUBs in hijacking host immune systems remains unknown. ORF64^USP^ is the tegument protein of Kaposi's sarcoma‐associated herpesvirus (KSHV). ORF64^USP^ was found to process K48‐ and K63‐linked polyubiquitin chains in vitro, and to target and deubiquitinate RIG‐I, thus leading to an inhibition of IFN‐I signaling.^[^ ^252^, ^254^ ^]^ HBx, encoded by hepatitis B virus, another DNA virus, deubiquitinates both RIG‐I and TRAF3 for K63‐linked ubiquitin chains and inhibits the production of type I interferon.^[^ ^255^, ^256^ ^]^ Epstein‐Barr virus (EBV) is a human oncogenic herpesvirus that presents a prolonged latent infection in the host. It has been found that EBV‐encoded BPLF1 interacts with and deubiquitinates TRAF6, inhibiting NF‐*κ*B signaling, and leads to viral lytic DNA replication upon lytic infection.^[^ ^257^ ^]^ BPLF1 was also shown to inhibit TLR signaling via the deubiquitination of downstream signaling components, such as NEMO and I*κ*B*α*.^[^ ^258^ ^]^

In addition to DNA viruses, RNA viruses have also evolved DUBs that involve in virus‐host interplay. Coronaviruses (CoVs) are a large family of RNA viruses that cause respiratory tract infections, ranging from the mild common cold to serious diseases such as severe acute respiratory syndrome (SARS), middle east respiratory syndrome (MERS), and the recent outbreak of the novel coronavirus, coronavirus disease 2019 (COVID‐19). These three diseases result from SARS‐CoV, MERS‐CoV, and SARS‐CoV‐2, respectively. Both SARS‐CoV and MERS‐CoV encode a papain‐like protease (PLP), featuring both deubiquitinating and deISGylating activities, which subsequently blocks the innate immune signaling of the proinflammatory cytokines.^[^ ^259^, ^260^ ^]^ Several studies have demonstrated that both SARS PLP and MERS PLP function as IFN antagonists by abolishing the phosphorylation and nuclear translocation of IRF3.^[^ ^259^, ^261^, ^262^ ^]^ Moreover, SARS PLP disrupts the interaction between the components in the STING‐TRAF3‐TBK1 complex and reduces the levels of ubiquitination of RIG‐I, STING, TRAF3, TBK1, and IRF3 in this complex, which yields a countermeasure against host antiviral immunity.^[^ ^263^ ^]^ Hence, both SARS‐CoV PLP and MERS‐CoV PLP can be promising therapeutic targets. Moreover, X‐ray structural analysis and biochemical assays have shown that both proteases share similar structures with the USP family DUBs.^[^ ^264^, ^265^, ^266^ ^]^ Despite the similarity, the differences between SARS PLP and MERS PLP lie in the recognition and cleavage specificities of the polyubiquitin chains. Specifically, SARS PLP prefers to cleave K48‐linked polyubiquitin chains, whereas MERS PLP shows no preference; SARS PLP adopts a bivalent mechanism of binding, namely by sensing a di‐Ub moiety as a minimal recognition element, in contrast with MERS PLP that cleaves one ubiquitin at a time.^[^ ^267^, ^268^ ^]^ Based on these findings, it is conceivable that the newly emerging SARS‐CoV‐2 also encodes DUBs to modify host innate immune responses, thus contributing to viral infection and pathogenesis. Another coronavirus is HCoV‐NL63, for which the DUB, HCoV‐NL63 PLP2, targets a set of signaling molecules: RIG‐I, TBK1, IRF3, and STING. This leads to the negative regulation of antiviral defenses that occur by disrupting STING‐mediated IFN induction.^[^ ^269^ ^]^ Mouse hepatitis virus (MHV) encoded PLP2 was reported to strongly inhibit cellular type I interferon expression. Further investigation revealed that MHV PLP2 achieves this by targeting both IRF3 and TBK1, and affects both activities.^[^ ^270^, ^271^ ^]^ Additional CoV PLPs include Porcine epidemic diarrhea virus (PEDV) replicase encoded PLP2, which was identified as an antagonist to both RIG‐I‐ and STING‐mediated IFN signaling by the reduction of RIG‐I and STING ubiquitination via its deubiquitinating activity that cleaves both K48‐ and K63‐linked polyubiquitin chains.^[^ ^272^ ^]^

In addition to coronaviruses, several other RNA virus‐encoded DUBs identify RIG‐I as solid targets. These DUBs are encoded by the Equine arteritis virus (EAV), porcine reproductive and respiratory syndrome virus (PRRSV), simian hemorrhagic fever virus (SHFV), and lactate dehydrogenase‐elevating virus (LDV), all belonging to the arterivirus family, as well as the Crimean‐Congo hemorrhagic fever virus (CCHFV), belonging to the nairovirus family.^[^ ^273^ ^]^ Previous studies have reported that the arterivirus‐ and nairovirus‐encoded DUBs resemble the OTU family of DUBs, and in the case of arteriviruses, the domain was characterized as PLP2.^[^ ^274^ ^]^ Notably, PRRSV‐encoded PLP2 also targets I*κ*B*α*.^[^ ^275^ ^]^ The foot‐and‐mouth disease virus (FMDV) is among the picornavirus family. Its product, L^pro^, targets not only RIG‐I, but also TBK1, TRAF3, and TRAF6, thereby collectively suppressing the host innate immune signaling pathways.^[^ ^276^ ^]^ For the Seneca Valley virus (SVV), another picornavirus, a study has shown that SVV 3C protease (3C^pro^) harbors deubiquitinating activity and acts on both K48‐ and K63‐linked polyubiquitin chains. The 3C^pro^ deubiquitinates RIG‐I, TBK1, and TRAF3, and consequently abolishes IFN‐*β* induction and IFN stimulated gene 54 (ISG54) mRNA expression.^[^ ^274^, ^277^ ^]^ We have listed these viral DUBs in Table 2.

Although it has been shown that various viral DUBs regulate the same target protein or pathway as their host cell counterpart, these virus‐encoded DUBs share no sequence or structural homology, which reflects the diversity and complexity of host‐virus interplay.^[^ ^278^, ^279^ ^]^ M1‐linked ubiquitination is indispensable in antiviral responses elicited by hosts. Recently, a unique M1‐linked ubiquitin chain‐specific effector DUB was identified in pathogenic bacteria; however, whether viral pathogens have evolved DUBs to cleave M1‐linked ubiquitin chains remains a question to be answered.^[^ ^280^ ^]^ The interactions between hosts and invading viruses are an emerging research field that provides deeper insights into viral pathogenic mechanisms. Thus, further research to elucidate the viral manipulation of DUBs is of particular importance.

## 6 DUBs as Therapeutic Targets in Innate Antiviral Immunity

Given the important roles of DUBs in innate antiviral immune response, and since DUBs harbor a catalytic domain or active center functioning as the target of inhibitors, it is not surprising that DUBs will be considered as potential therapeutic targets for a wide range of diagnoses and treatments.

In emerging basic studies, DUBs have shown promising prospects as drug targets for infectious diseases.^[^ ^33^ ^]^ The compound IU1, a reversible small‐molecule inhibitor of USP14, was shown to target the USP14 catalytic site.^[^ ^281^ ^]^ Functional assays revealed that IU1 inhibits replication of several flaviviruses, especially the Dengue virus.^[^ ^281^, ^282^ ^]^ Similarly, WP1130, directly inhibiting the DUB activity of USP5, UCH‐L1, USP9x, USP14, and UCH37,^[^ ^283^, ^284^ ^]^ shows broad‐spectrum antiviral activity for several RNA viruses.^[^ ^285^, ^286^ ^]^ Evidence showed that WP1130 inhibits USP9x activity through covalent modification of critical cysteine residues important for USP9x catalytic activity.^[^ ^287^ ^]^ PR‐619 is a general DUB inhibitor that nonetheless does not target other cysteine proteases like cathepsin B or calpain 1.^[^ ^288^ ^]^ In addition, P22077 was shown to covalently modify the catalytic cysteine of USP7 and induce a conformational switch in the enzyme associated with active site rearrangement, thus impairing HIV Gag processing and reducing the infectivity of released virions.^[^ ^289^, ^290^, ^291^ ^]^

As mentioned above, viral DUBs play pivotal roles in the regulation of host cellular processes, which provides us with novel insights into the possibility and validity of developing inhibitors that target the viral DUBs rather than the host DUBs. Indeed, several studies support the idea that PLP inhibitors may be effective in reducing coronaviruses infection.^[^ ^264^, ^292^, ^293^, ^294^ ^]^ To illustrate, GRL‐0617 was shown to be a potent, selective, and competitive noncovalent inhibitor of SARS‐CoV PLP.^[^ ^264^ ^]^ Several compounds, both clinical and preclinical, targeting SARS‐CoV PLP have been previously reviewed.^[^ ^295^ ^]^ The worldwide outbreak of SARS‐CoV‐2 compelled researchers to struggle with countermeasures to fight infection and prevent the spread of the virus. Since PLP inhibitors may be effective in reducing coronavirus infections, we propose that PLP inhibitors should be considered as a latent therapeutic strategy for COVID‐19. Recently, sequence analysis showed that the PLP present in SARS‐CoV‐2 has a high degree of similarity to that in the extensively studied SARS‐CoV.^[^ ^296^, ^297^ ^]^ Given that many active compounds against SARS‐CoV PLP have been clearly identified, evaluation of these inhibitors in targeting SARS‐CoV‐2 PLP should be put on the agenda. Intriguingly, several recent studies demonstrated that GRL‐0617 not only inhibits SARS‐CoV PLP but also inhibits the activity of SARS‐CoV‐2 PLP, thus impairing the virus‐induced cytopathogenic effect, facilitating the antiviral signaling, and reducing viral replication in infected cells.^[^ ^298^, ^299^ ^]^

Despite the promising insights on targeting both host and viral DUBs as an antiviral strategy, challenges exist in the following aspects. First, as previously mentioned in this review, DUBs function in delicate mechanisms with complexity. For instance, different DUBs may target the same substrate, while some may target more than one substrate. DUBs may perform opposing roles depending on the context. As an example, USP7 would be a plausible target in the oncology context, but inhibiting USP7 may have a potential impact on inflammatory responses. Hence, given the complexity and pleiotropy of DUBs, comprehensive approaches and diverse strategies are needed to develop these inhibitors. Second, there are still many unknowns about how DUBs work and are modulated. To date, many studies merely link DUBs to their functions in vitro, which is unconvincing, as the bona fide DUB activity in vivo may not be reflected.^[^ ^300^ ^]^ Third, previous inhibitors against viruses were designed to inhibit viral replication and inherent survival.^[^ ^301^ ^]^ The targets of these inhibitors are essential proteins for virus replication, assembly, and survival, which are effective in killing viruses in a short time.^[^ ^302^, ^303^ ^]^ However, due to strong selection pressure, the virus mutates or evolves to obtain long‐term resistance.^[^ ^304^, ^305^, ^306^ ^]^ Therefore, how to improve antiviral drugs to counter drug resistance is a key point.

Nevertheless, based on the pivotal role of DUBs in pathogenesis, the druggable feature, and the existing positive evidence against viral infection, developing inhibitors targeting DUBs, especially viral DUBs, should not be underestimated and might deserve at least a further evaluation.

## 7 Concluding Remarks

**Figure 7:**
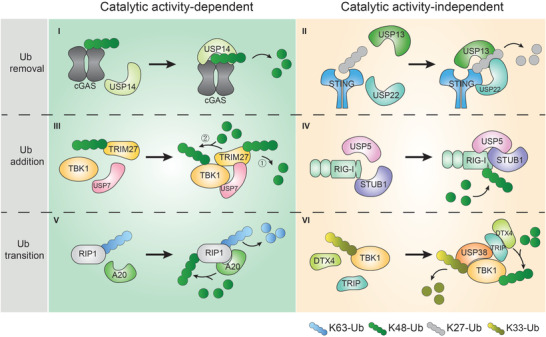
Schematic overview of six working models of DUBs in modulating immune responses. The underlying mechanisms of DUB function in innate immunity can be divided into six working models based on the pattern of alteration of the ubiquitination status.

As a key regulator of the ubiquitin system, DUBs play a pivotal role in antiviral innate immunity at different layers. Generally, DUBs act alone or in a complex to strengthen their catalytic activities. Specifically, the mechanisms underlying the function of DUBs in the antiviral signaling pathway can be divided into six working models, according to the pattern of altering the ubiquitination status (**Figure** **7**): i) most DUBs, such as USP14, cleave ubiquitin chains on their targets through catalytic activities; ii) several DUBs may remove ubiquitin chains independently of their enzymatic activities, such as USP22 which reduces ubiquitination by recruiting another molecule, USP13; iii) some DUBs may augment ubiquitination through their enzymatic activities, such as USP7 which stabilizes TRIM27 and thus leads to increased ubiquitin chains on TBK1; iv) certain DUBs may enhance ubiquitination in a protease‐independent manner, such as USP5 which recruits STUB1 to boost ubiquitination on RIG‐I, thereby leading to its degradation; v) DUBs such as A20 can reduce K63‐linked ubiquitination and simultaneously promote K48‐linked ubiquitination on RIP1 by itself via its DUB and E3 ligase activity, respectively; vi) DUBs such as USP38 may mediate ubiquitination transition by engaging other E3 ligases.

The effects of DUBs are intricate. While some DUBs positively regulate antiviral pathways, others may function as negative regulators. Still, some DUBs play dual roles (either inhibitory or activating) depending on specific contexts, such as subcellular localization or the type of substrate proteins. Some even act on identical substrates with opposing outcomes (such as USP4 and USP15).

Significant efforts have been dedicated to unveiling the virus‐encoded DUBs that are responsible for hijacking the cellular ubiquitin system to counteract host defense and evade host surveillance. While every effort was made to include in this Review all DUBs that have been identified, to date, in innate antiviral immunity, many more undoubtedly remain to be characterized. Many studies included in this Review relied on molecular and cellular approaches, which fail to accurately reflect the authentic circumstances that occur in vivo. Hence, further research is needed to ascertain the cellular targets and function of DUBs with in vivo animal experiments.

Given the growing evidence showing the importance of DUB activity in innate antiviral immunity, it is reasonable to consider DUBs as promising drug targets for infectious disease treatment. Such strategies should focus on tightly regulating the antiviral immune responses and enhancing infected cell death by modulating the DUBs involved. Additionally, virus‐encoded DUBs may provide attractive therapeutic targets for antiviral therapy. Given the importance and urgency of finding treatments for COVID‐19, the recommendation to evaluate potential drugs targeting SARS‐CoV‐2 PLP should not be underestimated. Overall, given the success of DUB inhibitors for cancer therapies, it is promising that the development of small‐molecule inhibitors or agonists targeting DUBs is on the right path.

There are still many gaps in our current knowledge and understanding of DUB mechanisms, which hinders progress in the therapeutic application of research in this area. First, unlike the designated roles of the canonical K63‐ and K48‐linked polyubiquitin chains, some atypical linkage types of polyubiquitin chains may exhibit quite distinctive roles. Yet, these atypical polyubiquitin chains are rarely reported in innate antiviral immune signaling. Thus, further research endeavors to elucidate the atypical polyubiquitin chains recognized by DUBs are required. Second, the activation and inhibition of key signaling molecules are determined by both E3 ligases and DUBs. How these E3 ligases and DUBs cooperate in a dynamic, temporal, and spatial manner to strictly regulate the immune response needs to be clarified further and deeper. Moreover, compared with the vast number of human DUBs, the number of virus‐encoded DUBs in control of host immunity appears limited. Therefore, identifying more viral DUBs that are responsible for modulating host immune system warrants increasing efforts to propel this field forward.

## Conflict of Interest

The authors declare no conflict of interest.

## Author Contributions

Z.Z., Z.K.Z., and L.W. contributed equally to this work. Z.Z. and Z.K.Z. conceived and drafted the manuscript. Z.Z. drew the figures. L.W. discussed the concepts of the manuscript. F.Z. and L.Z. provided valuable discussion and revised the manuscript. The authors apologize to those researchers whose related work were not able to cite in this review.
